# Rising stroke burden in Uganda: A national time-series analysis and forecasts, 2016–2028

**DOI:** 10.1371/journal.pone.0360963

**Published:** 2026-10-16

**Authors:** Suzan Nakasendwa, Joan Nankya Mutyoba, Alex Riolexus Ario, Evans Omondi, Brenda Birungi Bashemera, Francis Kakooza, Letisha Najjemba, Joyce Nakatumba Nabende, David Guwatudde, Agnes Kiragga

**Affiliations:** 1 Department of Epidemiology and Biostatistics, School of Public Health, Makerere University, Kampala, Uganda; 2 Uganda National Institute of Public Health, Ministry of Health, Kampala, Uganda; 3 African Population and Health Research Centre, Nairobi, Kenya; 4 Institute of Mathematical Sciences, Strathmore University, Nairobi, Kenya; 5 College of Computing and Information Sciences, Makerere University, Kampala, Uganda; 6 Infectious Diseases Institute, Makerere University, Kampala Uganda; 7 Committee on Data of the International Science Council – CODATA, Paris, France; Cedars-Sinai Medical Center, UNITED STATES OF AMERICA

## Abstract

**Introduction:**

Stroke is a leading cause of mortality and disability globally, with a disproportionate and growing burden in low- and middle-income Countries. However, evidence on national temporal trends and potential impacts in Uganda remains limited. This study examined trends in stroke cases and deaths in Uganda and forecast of future stroke burden through December 2028.

**Methods:**

We conducted a retrospective time-series analysis of District Health Information System (DHIS2) from 2016 to 2025. Monthly stroke cases, and deaths were analyzed. Monthly populations of Uganda were estimated using exponential growth interpolation and incorporated as an offset term in Bayesian negative binomial regression models to estimate incidence rate ratios (IRR) at 95% confidence interval (CI). Decomposition analysis using the Kitagawa method was applied to derive contributions from population growth and epidemiological change, based on model estimates. Stationarity and autocorrelation were assessed using the unit root and Ljung–Box tests. Forecasting was performed using the exponential smoothing state-space (ETS) model at 80% prediction intervals (PI).

**Results:**

Trend analysis showed sustained monthly increases in stroke incidence and mortality with IRR; 1.005 (95% CI: 1.004–1.006), and 1.006 (95% CI: 1.005–1.007), respectively (all p < 0.001). The epidemiological effect was approximately twice as large as the population effect, indicating that the majority of the increase in stroke cases and deaths is attributable to rising incidence rates rather than population growth alone. ETS model demonstrated that by December 2028, monthly cases and deaths are projected to reach approximately 927 (80% PI: 709–1,156) and 126 (80% PI: 94–160), respectively.

**Conclusion:**

Stroke burden in Uganda is projected to continue increase further over the next years in the absence of effective prevention measures. Strengthened primary prevention, expanded acute stroke care capacity, and improved surveillance systems are urgently needed to mitigate the growing health system impact.

## Introduction

Globally, stroke is the second leading cause of death and a major contributor to disability-adjusted life years (DALYs) lost [1]. Estimates from the Global Burden of Disease Study 2021 indicate that stroke accounted for approximately 7.3 million deaths and over 160 million DALYs globally, reflecting its substantial contribution to both premature mortality and long-term disability [2]. The burden of stroke is disproportionately concentrated in low- and middle-income countries (LMICs), accounting for nearly 70% of stroke deaths and 87% of stroke-related disability [3]. Although some high-income countries have experienced declines in age-standardized stroke mortality, many LMICs continue to face increasing absolute numbers of stroke cases and deaths due to a combination of population growth, ageing, and rising exposure to modifiable risk factors such as hypertension, diabetes, and unhealthy lifestyles [2,4].

In Sub-Saharan Africa (SSA), the burden of stroke has escalated dramatically over recent decades, driven by the epidemiological transition, rapid urbanization lifestyle changes, and the increasing prevalence of cardiovascular disease risk factors [5,6]. Population-based studies have documented crude annual incidence rates ranging from 25 per 100,000 in Nigeria, to approximately 260 per 100,000 in Egypt [7]. These differences highlight the considerable geographical and methodological heterogeneity in estimates of stroke burden across Africa. Despite this growing burden, the epidemiology of stroke in SSA remains incompletely characterized due to limitations in data availability and representativeness [8]. This phenomenon underscore the importance of strengthening population-based and routine surveillance systems, including District Health Information System 2 (DHIS2), to improve country-specific estimates [9].

Existing literature, often national planning estimates on stroke in SSA are commonly based on hospital-based cohorts, or on small geographic populations, or cross-sectional studies. In Uganda, stroke prevalence and incidence data remain limited, but available evidence suggests substantial and increasing burden [10,11]. Hospital-based studies have reported high case-fatality rates of 23.3% [12] and a 6% increased risk of mortality within 30 days of hospital admission [13]. However, such studies provide limited insight into national-level trends, the temporal dynamics and long-term trajectories of stroke burden [14].

Routine health information systems, such as the DHIS2 have emerged as the predominant health management information system platform in LMICs, currently deployed at national scale in over 30 Countries and supporting health service delivery [15,16]. DHIS2 enables systematic collection, aggregation, and analysis of routine health facility-based data across multiple health programs [16]. In Uganda, DHIS2 has been utilized for disease surveillance and health program monitoring, including for COVID-19 response, demonstrating the platform’s flexibility and local capacity for rapid adaptation [9]. However, challenges related to data quality, completeness, and timeliness have been documented across DHIS2 implementations in SSA, necessitating careful preprocessing and validation when utilizing these data for epidemiological analysis and forecasting [17,18].

Despite the potential of DHIS2 data to provide population-level insights, these data remain underutilized for longitudinal analyses and forecasting applications [19,20]. Time-series analysis and forecasting methods have been successfully applied to predict healthcare utilization and disease burden across various conditions [21,22]. Despite the growing recognition of stroke as a priority non-communicable disease in Uganda, comprehensive analyses of national trends and future projections remain limited. This study examined trends in stroke cases, and stroke-related deaths, using routinely collected national data between January 2016 and December 2025. We further projected stroke events beyond the year 2025, through December 2028 using time-series forecasting methods.

## Materials and methods

### Study design and setting

This study employed a retrospective study design using routinely collected stroke data in Uganda, to examine temporal trends using time-series analysis, and to forecast stroke events. Data were obtained from the national District Health Information System (DHIS2) of Uganda, which compiles routine service delivery data reported by health facilities throughout the country. Because annual reporting of stroke events in DHIS2 is available from the year 2016 onwards, monthly counts for each indicator were extracted for the period January 2016 to December 2025. Annual population data of Uganda from the year 2015–2026 were obtained from Worldometer, which reports population estimates derived from the United Nations Department of Economic and Social Affairs, Population Division [23].

### Stroke events and definitions

Data were extracted from the DHIS2 platform and included three routine service indicators related to stroke. The variable “*105-CV01. Stroke/Cerebrovascular Accident (CVA)”* represents the total number of patients recorded with a clinical diagnosis of stroke (cerebrovascular accident) during the reporting period, reflecting overall stroke-related service utilization or admissions. The variable “*108-CV03a. Stroke/Cerebrovascular Accident (CVA) - Cases”* captures the number of confirmed stroke cases documented within the same period, as reported by health facilities through routine surveillance registers. The variable *“08-CV03b. Stroke/Cerebrovascular Accident (CVA) - Deaths”* denotes the number of in-facility deaths among patients diagnosed with stroke during the reporting period.

All variables were aggregated at the reporting-unit level and represent routinely collected administrative data. For clarity and consistency in the manuscript, these indicators were relabeled as *suspected stroke cases*, *confirmed stroke cases*, and *stroke deaths*, respectively, while preserving their original operational definitions as defined in DHIS2.

### Data management

All data were cleaned, prepared and analyzed using R programming language, version 4.5.2. We extracted stroke monthly data per year per stroke event, creating a dataset of five variables (Year, month, suspected stroke cases, confirmed stroke cases, stroke deaths). Variables; year and month were combined to create a continuous monthly date variable “period” with values 2016-Jan to 2025-Dec. Annual population figures as of December 2015 to December 2025 were obtained.

Because stroke events data was extracted on a monthly basis, annual population figures were interpolated to generate monthly population estimates. Interpolation was performed assuming exponential population growth, reflecting a gradual and continuous demographic change over time. The annual population as of December 2015 was only used to generate the population exponential growth rate from December 2015 to December 2016. This growth rate was used to interpolate monthly populations from January 2016 to November 2016. However, the annual population in 2015 was not used in the trends analysis. A similar exponential interpolation strategy was implemented for the years 2016–2025 to generate monthly populations. This set of data consisted of the date variable “period” and monthly population. The stroke, and population datasets were then merged into the analyzable dataset of five variables (period, suspected stroke cases, confirmed stroke cases, stroke death, and population), using “period” as the unique identifier.

### Statistical analysis

#### Data pre-processing.

Time-series plots were explored to identify temporal patterns in suspected stroke cases, confirmed stroke cases, and stroke deaths. Outliers were identified using the tso() function, with autoregressive integrated moving average (ARIMA) model parameters automatically selected via auto.arima() from the forecast. The procedure detects statistically significant additive outliers based on residual diagnostics from the fitted ARIMA model. Identified outliers were replaced with ARIMA model adjusted values, and the resulting cleaned data was used for subsequent trend analyses and forecasting.

The seasonal and Trend decomposition using Loess (STL) was applied to assess the strength of seasonality and trend. Seasonal subseries plots were used to assess within-year variation by displaying monthly stroke counts across all years. Seasonality was evaluated by visually examining whether specific months consistently showed higher or lower values and whether recurring monthly patterns were evident.

#### Descriptive analysis of stroke rates.

Suspected stroke cases were retained for descriptive purposes and to assess trends in case detection. However, all primary analyses including incidence, mortality, decomposition and forecasting were based on confirmed stroke cases.

For each month, we calculated the incidence rate as the number of confirmed stroke cases per 100,000 population, and the mortality rate as the number of stroke deaths per 100,000 population. The case fatality rate (CFR) was computed as the proportion of confirmed stroke cases resulting in death.

To illustrate temporal trends, we generated line graphs of incidence, mortality, and CFR over the study period. These visualizations facilitated the identification of long-term trends, seasonal patterns, and potential fluctuations in reporting or disease occurrence. Graphical inspection of the trends also informed subsequent modeling and decomposition analyses.

#### Trends analysis.

Formal trend analysis was conducted using Bayesian negative binomial regression models to account for over-dispersion in monthly stroke counts, while enabling probabilistic inference through posterior distributions and uncertainty quantification. Time, measured in months, was included as a continuous predictor to estimate temporal trends.

To account for changes in population size over time, monthly interpolated population estimates were included as an offset term in all regression models. The natural logarithm of the population was specified as the offset, allowing model coefficients to be interpreted as rates rather than crude counts. Exponentiated time coefficients are reported as incidence rate ratios (IRR) representing the average monthly change in stroke rates. Model adequacy was assessed through residual diagnostics using autocorrelation function plot. Statistical significance was evaluated at the 5% level, and results were reported with 95% confidence intervals.

#### Decomposition of stroke burden.

To quantify the drivers of changes in stroke burden over time, we performed a decomposition analysis using model-based estimates. Model predicted counts were aggregated to annual totals, and corresponding rates per 100,000 population were computed using annual population estimates. These model-based estimates were then used in a decomposition framework to separate the observed changes in stroke cases and deaths into two components: (1) a population effect, representing changes attributable to population growth, and (2) an epidemiological effect, representing changes in stroke incidence and mortality independent of population size.

We applied the Kitagawa decomposition method [24], a symmetric approach widely used in epidemiology and demography [25,26], which decomposes year-to-year changes by averaging the rates and population sizes of consecutive years. This approach minimizes bias associated with selecting a single baseline year and provides a balanced estimate of the contributions of population growth and changes in incidence and mortality. Decomposition results were expressed both as absolute changes in counts and as rates per 100,000 population to facilitate comparison with incidence and mortality trends.

To aid interpretation, the contributions of population and epidemiological effects were visualized using line graphs, illustrating their relative roles in driving temporal changes in stroke burden over the study period. This approach allowed us to distinguish whether increases in stroke burden were primarily driven by demographic expansion or by changes in the underlying risk of stroke.

#### Forecasting.

Prior to forecasting model fitting, stationarity was evaluated using the unit root test. Ljung-Box test and autocorrelation functions were examined to characterize temporal dependence and guide model specification. Data from 2016 to 2024 were used for model training, while 2025 was held out as a testing period to evaluate out-of-sample forecasting performance. Forecasts were then generated for 2025 and the subsequent period, 2026–2028. This three-year horizon was selected to capture multiple seasonal cycles while maintaining reasonable predictive reliability, thereby supporting strategic health service planning under the assumption of stable trends and reporting patterns.

Forecasting models were then fitted using Naive model as a baseline model, ARIMA and exponential smoothing state-space frameworks. Model selection was based on minimization of Root Mean Square Error (RMSE) and Mean Absolute Percentage Error (MAPE), with preference given to parsimonious models that adequately captured the underlying temporal structure of the data. Fitted values and forecasts, with prediction intervals were used to quantify uncertainty around the projected values. Forecast uncertainty was expressed using 80% prediction intervals to provide a narrower and more operationally relevant range for short-term planning. The 80% interval captures the most probable forecast values while avoiding excessively wide bounds that may reduce interpretability in health service planning contexts.

Forecast performance and plausibility were evaluated through comparison of fitted values with observed data in the training period, and through inspection of forecast trajectories to ensure epidemiological coherence.

## Results

### Population, stroke events and rates in Uganda from January 2016 to December 2025

The population in Uganda has steadily increased from approximately 37.5 million in 2015 to over 50 million in 2025. Between 2016 and 2025, the burden of stroke in Uganda increased substantially (Table 1). The annual number of confirmed stroke cases rose from 3,089 in 2016–8,449 in 2025, representing nearly a threefold increase over the study period. This corresponds to the increase in incidence rate per 100,000 population from 8.08 to 16.65.

**Table 1:** Annual population, stroke events and rates in Uganda from January 2016 to December 2025.

Year	Population	Suspected stroke cases	Confirmed stroke cases	Stroke deaths	Incidence per 100,000 population	Mortality per 100,000 population	Case fatality rate
2016	38,214,635	11,274	3,089	342	8.083	0.895	0.111
2017	39,541,715	10,804	3,970	412	10.040	1.042	0.104
2018	40,925,558	14,140	4,991	561	12.195	1.371	0.112
2019	42,338,452	15,404	5,878	584	13.883	1.379	0.099
2020	43,785,104	12,103	5,032	604	11.492	1.379	0.120
2021	45,240,744	15,697	5,683	702	12.562	1.552	0.124
2022	46,666,743	15,034	5,588	643	11.974	1.378	0.115
2023	48,037,540	17,697	6,893	790	14.349	1.645	0.115
2024	49,389,355	18,075	7,866	928	15.927	1.879	0.118
2025	50,754,005	22,645	8,449	908	16.647	1.789	0.107

Stroke related deaths also increased from 342 to 908, with the mortality rate rising from 0.90 to 1.79 per 100,000 population (Table 1). Despite these increases, the case fatality rate remained relatively stable, fluctuating between 9.9% and 12.4%.

### Trends in stroke events from January 2016 to December 2025

Fig 1 panel A illustrates monthly trends in suspected and confirmed cases of Stroke over the study period. Suspected cases displayed marked short-term variability with recurrent peaks, but with an overall increasing trajectory from 2016 to 2025. Confirmed cases similarly showed a steady upward trend, with lower magnitude and less variability.

**Fig 1:**
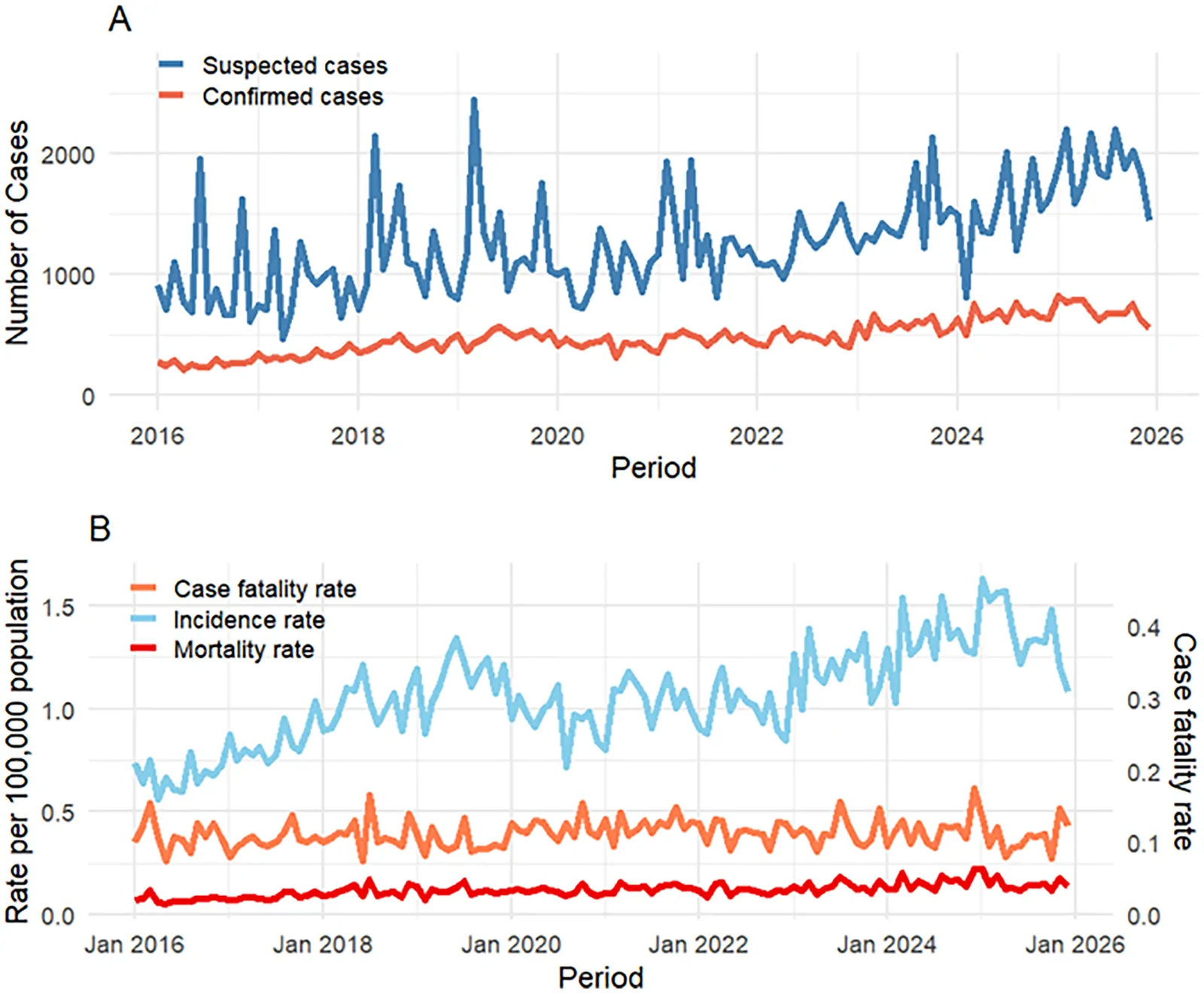
Time series plots of stroke events and rates from January 2016 to December 2025.

Fig 1 panel B presents temporal trends in incidence, mortality, and case fatality rates. The incidence rate demonstrated a sustained upward trend, indicating an increase in the population-level risk of stroke over time. Mortality rates also increased, though more gradually, suggesting a growing absolute burden of stroke-related deaths. In contrast, the case fatality rate fluctuated within a relatively narrow range without a consistent temporal pattern, indicating that the probability of death among confirmed stroke cases has remained relatively stable.

Unit root tests confirmed stationarity in confirmed stroke cases and stroke deaths with p-values of 0.01, indicating stable variance and mean after preprocessing (Table 2). The STL seasonal strength was lower than 0.64 cutoff for both series; 0.350 for confirmed cases and 0.247 for deaths indicating weak seasonal patterns. The seasonal series plots also indicated non seasonality of the stroke events (S1 Fig).

**Table 2:** Trend analysis estimates from a Bayesian negative binomial model, and corresponding data tests for the data from January 2016 to 2025.

Stroke indicator	Stationarity Unit root test, P-value	STL^a^ Seasonal strength	Trend analysis		Autocorrelation Ljung-Box test, P-value
			IRR (95% CI)	P-value	
**Confirmed stroke cases**	0.01	0.350	1.005 (1.004, 1.006)	<0.001	0.2943
**Stroke deaths**	0.01	0.247	1.006 (1.005, 1.007)	<0.001	0.2091

^a^STL = Seasonal and Trend decomposition using Loess.

Confirmed stroke cases increased significantly over time (IRR: 1.005; 95% CI: 1.004–1.006; p < 0.001), corresponding to an average monthly increase of approximately 0.5% in cases rates per month after adjusting for population growth, short term and seasonal dependencies (Table 2). Stroke-related deaths demonstrated a consistent upward trend with an IRR of 1.006 (95% CI: 1.005–1.007; p < 0.001). These findings indicate that the observed increases reflect rising stroke rates rather than population expansion alone. The Ljung-Box test results showed no evidence of significant residual autocorrelation for confirmed cases and deaths (both P > 0.05).

### Stroke burden decomposition: Population growth versus epidemiological change

The decomposition analysis shows that increases in both confirmed stroke cases and stroke deaths were driven by contributions from population growth and changes in underlying rates; however, the rate (epidemiological) effect consistently contributed more than the population effect throughout the study period (Table 3).

**Table 3:** Model-based decomposition estimates for annual stroke incidence and mortality from 2016 to 2025.

Confirmed stroke cases							
**Year**	**Population**	**Predicted cases**	**Model-based rate**	**Population effect**	**Rate effect**	**Population effect rate**	**Epidemiological effect rate**
2016	38,214,635	3650.4	9.55	–	–	–	–
2017	39,541,715	4017.7	10.16	130.8	236.5	0.331	0.598
2018	40,925,558	4405.8	10.77	144.8	243.3	0.354	0.595
2019	42,338,452	4848.2	11.45	156.9	285.4	0.371	0.674
2020	43,785,104	5327.6	12.17	170.8	308.6	0.390	0.705
2021	45,240,744	5844.2	12.92	182.6	334.0	0.404	0.738
2022	46,666,743	6408.9	13.73	190.0	374.6	0.407	0.803
2023	48,037,540	6990.2	14.55	193.9	386.5	0.404	0.807
2024	49,389,355	7626.6	15.44	202.7	433.7	0.410	0.878
2025	50,754,005	8331.8	16.40	217.3	479.9	0.428	0.946
**Stroke deaths**							
**Year**	**Population**	**Predicted deaths**	**Model-based rate**	**Population effect**	**Rate effect**	**Population effect rate**	**Epidemiological effect rate**
2016	38,214,635	398.1	1.04	–	–	–	–
2017	39,541,715	440.2	1.11	14.3	27.8	0.036	0.070
2018	40,925,558	488.5	1.19	16.0	32.3	0.039	0.079
2019	42,338,452	539.5	1.27	17.4	33.5	0.041	0.079
2020	43,785,104	595.7	1.36	19.1	37.2	0.044	0.085
2021	45,240,744	658.4	1.46	20.5	42.1	0.045	0.093
2022	46,666,743	724.9	1.55	21.5	45.1	0.046	0.097
2023	48,037,540	797.1	1.66	22.0	50.2	0.046	0.105
2024	49,389,355	876.7	1.78	23.2	56.4	0.047	0.114
2025	50,754,005	963.1	1.90	25.1	61.4	0.049	0.121

For confirmed stroke cases, both the population effect and the rate effect increased progressively from 2017 to 2025. The population effect rose from 130.8 to 217.3 cases, corresponding to an increase from 0.331 to 0.428 per 100,000 population. In contrast, the rate effect increased more sharply, from 236.5 to 479.9 cases, with the corresponding epidemiological effect rate rising from 0.598 to 0.946 per 100,000 (Fig 2). Across all years, the epidemiological effect was approximately twice as large as the population effect, indicating that the majority of the increase in stroke cases is attributable to rising incidence rates rather than population growth alone.

**Fig 2:**
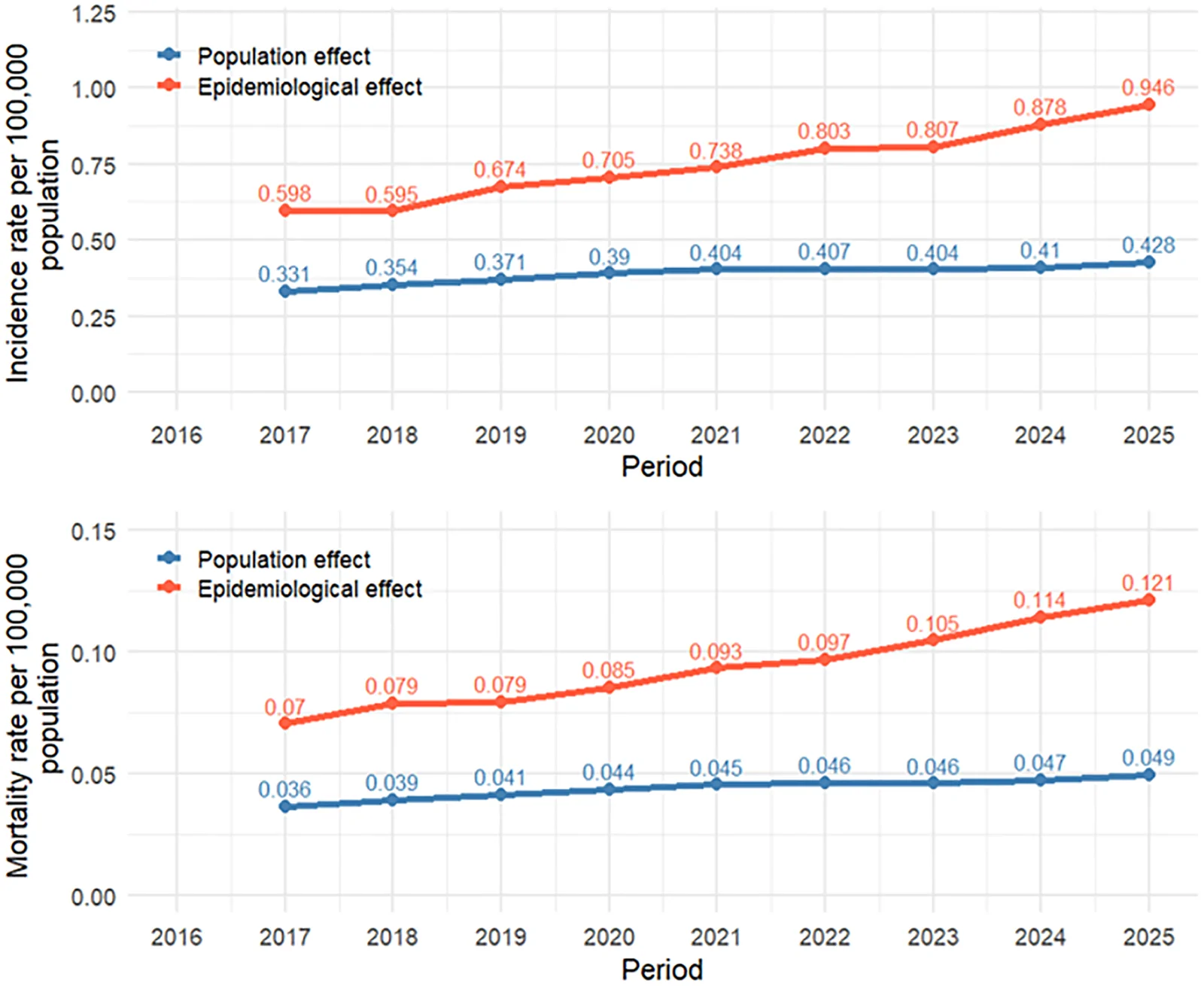
Trend in model-based annual decomposition estimates of stroke incidence and mortality from 2016 to 2025.

A similar pattern was observed for stroke deaths. The population effect increased modestly from 14.3 to 25.1 deaths (0.036 to 0.049 per 100,000), while the rate effect increased more substantially from 27.8 to 61.4 deaths (0.070 to 0.121 per 100,000) (Table 3, Fig 2). As with stroke cases, the epidemiological effect consistently exceeded the population effect across all years, demonstrating that increases in stroke mortality were primarily driven by rising mortality rates rather than demographic expansion.

Overall, these findings indicate that although population growth contributed to the increasing burden of Stroke, the dominant driver was the change in underlying incidence and mortality rates.

### Forecasting of stroke cases and deaths until December 2028

Forecast model evaluation demonstrated that exponential smoothing state-space (ETS) models provided the most accurate and parsimonious forecasts for both stroke cases and deaths (Fig 3). The comparative evaluation of forecasting models for Stroke cases indicated that both the exponential smoothing (ETS) and autoregressive integrated moving average (ARIMA) models outperformed the naive benchmark. The ETS model demonstrated the best performance, with lower prediction errors as reflected by the root mean squared error (RMSE = 50.61) and mean absolute percentage error (MAPE = 9.05). The ARIMA model showed comparable but slightly higher errors, while the naive model performed substantially worse (RMSE = 72.30; MAPE = 12.50), indicating its limited ability to capture temporal patterns in stroke cases.

**Fig 3:**
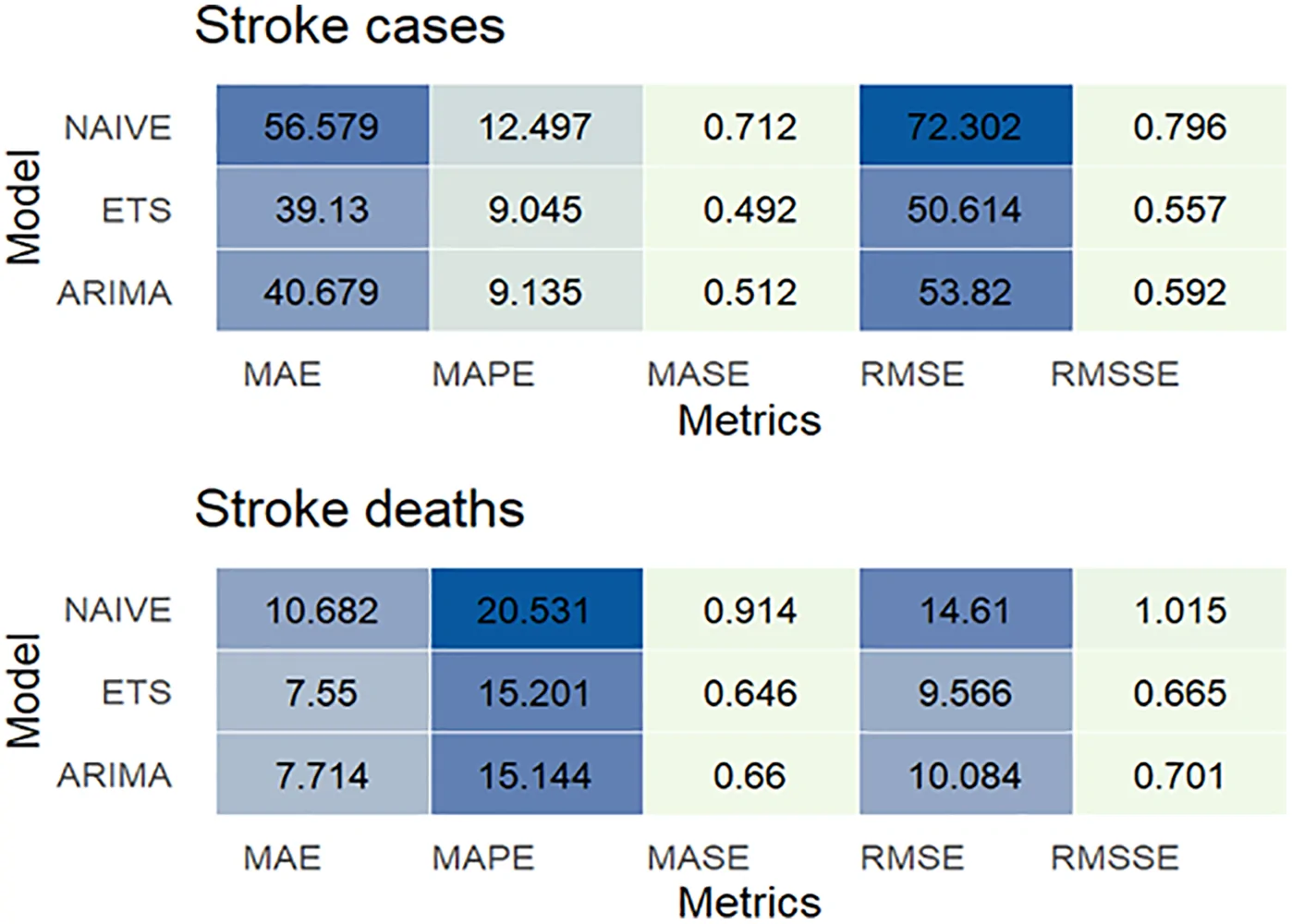
Model evaluation metrics from the three fitted models for forecasts until December 2028.

A similar pattern was observed for stroke deaths. The ETS model again yielded the lowest prediction errors (RMSE = 9.57; MAPE = 15.20), followed closely by the ARIMA model. In contrast, the naive model showed considerably poorer performance, particularly with respect to MAPE (20.53). These findings suggest that the ETS model, provide more accurate and reliable forecasts of stroke burden compared to simpler baseline approaches.

The forecasts indicate a continued increase in the burden of Stroke over the projection period. For stroke cases, the model predicts a sustained upward trend from 2025 onwards, with the number of cases expected to reach approximately 927 by December 2028. The associated 80% prediction interval (709−1,156) suggests moderate uncertainty around the point estimate, with the widening prediction intervals over time reflecting increasing uncertainty further into the forecast horizon. Despite this uncertainty, the trajectory remains consistently upward, indicating a likely continued rise in stroke incidence (Fig 4).

**Fig 4:**
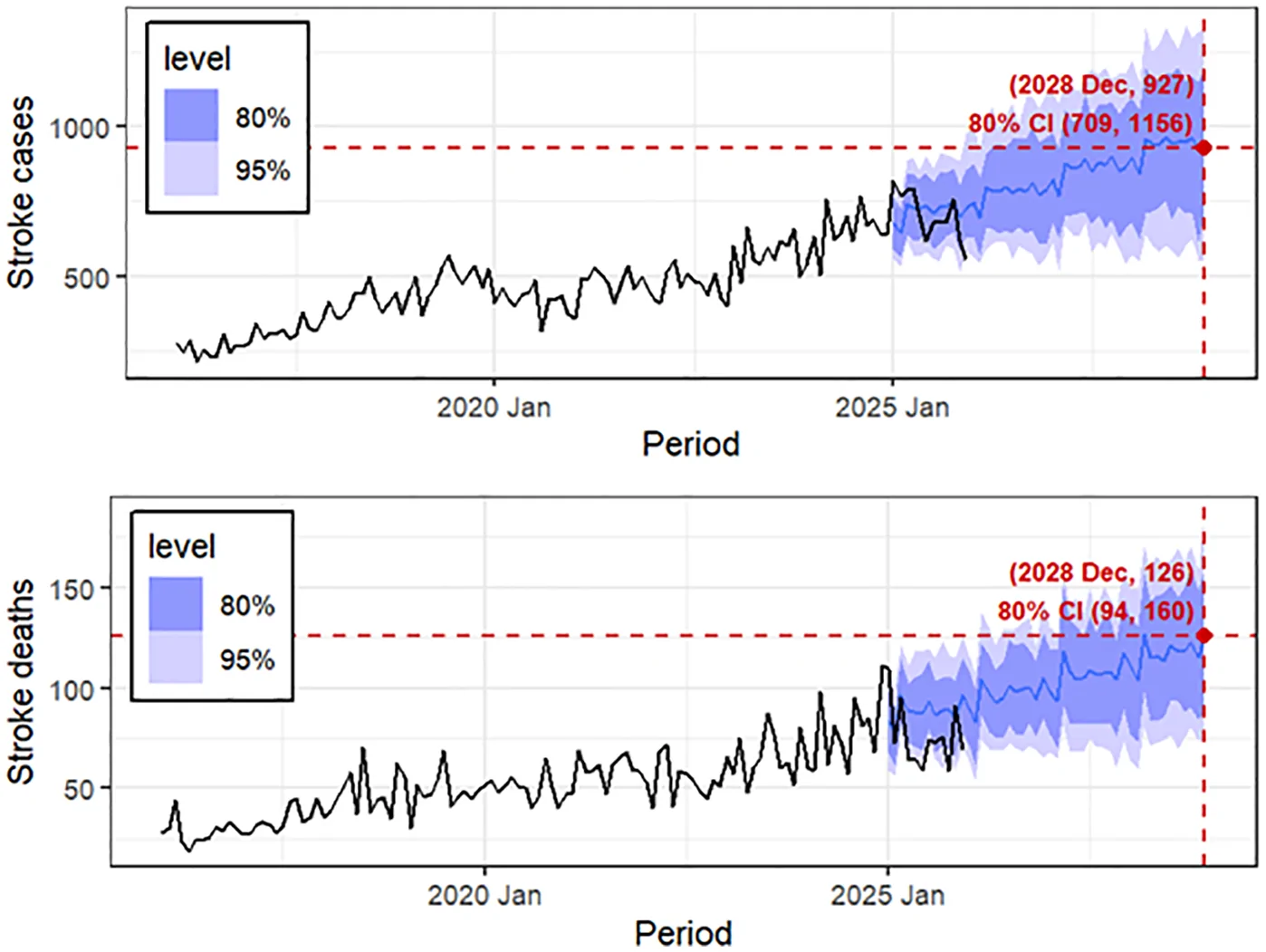
Forecast plot of the predicted trends from the exponential smoothing state-space (ETS) model until December 2028.

A similar pattern was observed for stroke deaths, with projections indicating an increase to approximately 126 deaths by December 2028 (80% prediction interval: 94–160). As with stroke cases, the forecast intervals widen over time, but the overall trend remains upward. These findings mean that, in the absence of effective interventions, both stroke incidence and mortality are expected to continue increasing in the coming years. The consistency between historical trends and projected patterns reinforces the robustness of the model and highlights the growing public health burden posed by stroke.

## Discussion

This study examined trends in stroke cases, and stroke-related deaths, using routinely collected national data between January 2016 and December 2025. Stroke cases, and deaths increased significantly over the years 2016–2025, even after adjusting for exponential population growth. Despite these increases, the case fatality rate remained relatively stable, fluctuating between 9.9% and 12.4%. Trend analysis showed sustained monthly increases, with incidence rate of 0.5% for confirmed cases, and mortality rate of 0.6%, indicating rising stroke rates beyond demographic expansion. The epidemiological effect was approximately twice as large as the population effect, indicating that the majority of the increase in stroke cases and deaths is attributable to rising incidence rates rather than population growth alone. Forecasting models projected continued growth through December 2028, with monthly stroke cases expected to reach approximately 927 cases per month, and deaths around 126 per month.

The decline in reported suspected stroke cases from 15,404 in 2019–12,103 in 2020 could be as a result of the COVID-19 pandemic. Although a true change in stroke occurrence cannot be excluded, the reduction may also reflect disruptions in healthcare access and utilization, changes in health-seeking behavior, and interruptions in routine health service delivery and DHIS2 data reporting during the pandemic [27,28]. Similar disruptions in healthcare utilization, service delivery, and routine disease reporting were also documented in several countries during the COVID-19 pandemic [29–31], supporting the possibility that the decline observed in Uganda was partly influenced by pandemic-related health-system disruptions.

The observed increase in reported stroke incidence between 2016 and 2025 may reflect both a rise in stroke burden and improvements in data capture, reporting completeness, health-facility coverage, and the overall capacity of the health information system over time [20,32,33]. However, these factors cannot be disentangled using the available routine data. The estimated stroke incidence of 16.65 per 100,000 population in 2025 represents the burden of stroke captured through hospital-based routine reporting. Previous research have noted that much of the available stroke epidemiological evidence in Africa is derived from hospital-based studies, which may underestimate the true population burden because patients with minor strokes may not seek hospital care, while those with rapidly fatal strokes may die before hospitalization [34]. Consistent with the study findings, hospital-based studies have reported substantial variation in stroke incidence, ranging from 26 per 100,000 in Nigeria [7] to 149 per 100,000 in Mozambique [35], while community-based studies have reported considerably higher estimates, including 181–250 per 100,000 in Egypt [36,37] and 108–316 per 100,000 in Tanzania [38]. The lower incidence observed in this study may reflect under-ascertainment of events that do not reach reporting health facilities.

These findings are consistent with the currently observed stroke situation across LMICs [39,40]. As Uganda undergoes rapid urbanization and lifestyle shifts, the prevalence of hypertension, diabetes, and obesity has surged [41], yet the health system remains largely oriented towards infectious disease management [42] with limited investment in services for non-communicable disease including stroke [43]. Research across SSA confirms that stroke incidence is rising among younger, working-age populations [7,44]. This demographic shift carries profound socioeconomic implications, as stroke-related disability in younger adults leads to significant loss of productivity and places immense financial strain on families who must provide long-term care without institutional support [45].

The decomposition findings align closely with evidence from the Global Burden of Disease (GBD) Study 2021, which shows that increases in the absolute number of stroke cases and deaths in many low- and middle-income countries are largely attributable to changes in age-specific incidence and mortality rates, alongside but not limited to demographic expansion [1]. While population growth and ageing contribute to the overall rise in stroke burden, GBD analyses highlight that persistent exposure to modifiable risk factors particularly hypertension, metabolic disorders, and lifestyle-related factors has played a central role in driving epidemiological transitions in sub-Saharan Africa [2,46,47]. The observed dominance of the rate effect in this study, where it contributed approximately twice as much as the population effect for both incidence and mortality, therefore supports the interpretation of a true increase in underlying stroke risk rather than a purely demographic phenomenon.

However, the upward trend in Uganda stands in sharp contrast to the stroke transition observed in many high-income countries (HICs). Over the last three decades, HICs have generally seen a decline in age-standardized stroke incidence and mortality, driven by mature primary care systems and the widespread use of antihypertensive and statin therapies [2]. For instance, while East Asian nations like Japan and South Korea have successfully reduced stroke rates through aggressive public health policy, the burden in India and China remains high due to aging and poorly controlled risk factors [46,48]. Africa and Uganda’s lack of a similar decline suggests that the preventive strategies benefiting HICs such as early screening are either under-resourced or less effective in this context [49,50].

Ultimately, our projections reinforce the World Stroke Organization Lancet Neurology Commission’s warning that without urgent intervention, global stroke deaths could rise by 50% by 2050, with 91% of those fatalities occurring in LMICs [51]. The upward trends observed in Uganda are consistent with this projected global shift toward a disproportionate stroke burden in LMICs [6,52,53]. To mitigate this trajectory, decentralization of hypertension management through integrated primary care models will be critical, alongside the establishment of “stroke-ready” units capable of delivering timely diagnosis, acute management, and early rehabilitation. Uganda has initiated programs of integrated care within primary care health facilities, but this will need to be coupled with strong community partnerships that support stroke care and prevention [54,55].

Strengthening surveillance systems to capture comprehensive stroke data, including community-level events, is equally important, as the facility-based data analyzed in this study likely reflects predominantly more severe cases and may underestimate the true burden. Future research could investigate stroke subtypes, vascular risk profiles, and care pathways specific to the Ugandan population to guide more targeted and contextually appropriate prevention strategies.

### Implications for practice

The projected increase in Stroke burden, coupled with the decomposition findings demonstrating a dominant epidemiological (rate) effect, suggests that future demand for stroke-related healthcare will be driven not only by population growth but, more importantly, by a sustained rise in underlying stroke risk. In the absence of effective prevention and health system strengthening, this trajectory is likely to translate into substantial increases in stroke incidence and mortality over the coming years. This has important implications for health system planning in Uganda, including the need to expand workforce capacity, improve access to diagnostic imaging, strengthen acute stroke care services, and scale up rehabilitation and long-term care. Health systems that are already resource-constrained may face considerable strain if service delivery is not scaled in parallel with the rising burden.

From a prevention perspective, the predominance of the epidemiological effect underscores the urgent need to prioritize population-level interventions targeting modifiable risk factors, particularly hypertension, diabetes, tobacco use, and unhealthy diets. Strengthening the integration of non-communicable disease screening and management within primary healthcare platforms is critical to reducing stroke incidence. In addition, enhancing routine surveillance systems that capture both facility-based and community-level stroke events would enhance the accuracy of burden estimates and support more responsive health planning. Without timely investment in prevention, acute care, and rehabilitation, the projected increase in stroke burden is likely to translate into escalating healthcare costs, productivity losses, and avoidable mortality.

### Limitations of the study

This study has several limitations. First, the analysis was based on routinely reported, facility-based DHIS2 data and therefore reflects reported stroke events rather than the complete population burden. Stroke events that did not result in presentation to or admission at a reporting health facility may have been missed, particularly in settings with limited access to healthcare, which may result in selection bias and underreporting of stroke events. Differences in access to diagnostic services, availability of specialized care, and care-seeking behaviors likely contributed to under-ascertainment in less-resourced settings and higher detection in urban areas. These factors may have biased the observed distribution of cases and limited the feasibility of hierarchical modeling due to sparse and imbalanced data.

Second, the study relied on routinely reported indicators and their corresponding DHIS2 reporting definitions. The data were analyzed according to the available indicator definitions and reporting structure; however, individual clinical records were not available to independently verify diagnoses, neuroimaging confirmation, or completeness of case ascertainment. Thus, misclassification, under-reporting, and changes in reporting completeness or diagnostic capacity over time cannot be completely excluded. Although outlier detection and correction were applied to mitigate the influence of anomalous observations, residual reporting biases cannot be fully excluded. The observed temporal increase may therefore reflect both changes in the underlying stroke burden and improvements in health-service utilization and reporting.

Third, while population offsets were used to account for demographic change, the interpolated population estimates are based on assumptions of exponential growth and may not fully capture short-term demographic fluctuations and therefore introduce some uncertainty into incidence estimates. Finally, forecasting inherently involves uncertainty, particularly over longer horizons. Although model diagnostics and evaluation metrics supported the selected forecasting models, projections should be interpreted as plausible future scenarios rather than precise predictions, especially in the context of potential future changes in population structure, health policy and service delivery, reporting practices, and stroke risk factors.

### Strengths of the study

This study has several important strengths. First, it uses a national routine health information dataset covering an extended period, enabling assessment of temporal and geographic patterns in reported stroke cases and deaths beyond the scope of individual hospital-based studies. Second, the analysis incorporates population denominators and accounts for over-dispersion and uncertainty in routinely reported count data, providing more robust estimates of stroke incidence and mortality.

Third, the decomposition analysis distinguishes the contributions of population growth from changes in the underlying incidence rate, providing a more informative interpretation of the increasing number of reported stroke events. Finally, the integration of temporal modelling and forecasting provides evidence on both historical patterns and potential future trends, supporting health-system planning and prioritization of stroke prevention and care services.

## Conclusion

Stroke represents an increasing public health burden in Uganda, with sustained increases in reported admissions, cases, and deaths over the study period. Rate-based analyses indicate that the observed extend beyond population growth alone, although improvements in case ascertainment and reporting may also have contributed. Forecasts suggest that the number of reported stroke events is likely to continue increasing through 2028 if the current patterns persist. These findings underscore the need to strengthen stroke prevention through integrated management of modifiable risk factors, particularly hypertension and other cardiovascular risks, while expanding timely access to acute stroke care and rehabilitation services. Strengthening routine stroke surveillance, improving the completeness and quality of DHIS2 reporting, and using surveillance data to guide resource allocation and service planning will be important for responding to the projected increase in stroke burden and reducing preventable deaths and disability.

## Supporting information

S1 Fig Seasonal sub-series plots of confirmed stroke cases and deaths from 2016 to 2025. (TIF)
